# Biochemical Alterations in Semen of Varicocele Patients: A Review of the Literature

**DOI:** 10.1155/2012/903931

**Published:** 2011-09-11

**Authors:** Antonio Mancini, Roberto Festa, Sebastiano Raimondo, Andrea Silvestrini, Elena Giacchi, Gian Paolo Littarru, Alfredo Pontecorvi, Elisabetta Meucci

**Affiliations:** ^1^Division of Endocrinology, Department of Internal Medicine, Catholic University of the Sacred Heart, Rome 00168, Italy; ^2^Department of Molecular Pathology, University of Marche, Ancona 60020, Italy; ^3^Institute of Biochemistry and Clinical Biochemistry, Catholic University of the Sacred Heart, Rome 00168, Italy; ^4^Centre for Study and Research on Natural Fertility Regulation, Catholic University of the Sacred Heart, Rome 00168, Italy; ^5^Department of Biology, Biochemistry and Genetics, University of Marche, Ancona 60020, Italy

## Abstract

Oxidative stress is a mechanism underlying different kinds of infertility in human males. However, different results can be observed in relation to the method used for its evaluation. Varicocele patients show a number of biochemical abnormalities, including an altered distribution of coenzyme Q between seminal plasma and sperm cells and also an apparent defect in the utilization of antioxidants. Moreover, an influence of systemic hormones on seminal antioxidant system was observed too. Finally, the effects of surgical treatment on oxidativestress indexes and the possible usefulness of some medical therapies, like coenzyme Q supplementation, are discussed. In conclusion, published data show a role of oxidative stress in varicocele-related male infertility, but at present we do not know the precise molecular mechanisms underlying these phenomena.

## 1. Oxidative Stress in Male Infertility

An excess of reactive oxygen species (ROS) and other oxidant radicals, in the body but in particular at genital level, has been associated with male infertility [1–5]. The high content of polyunsaturated fatty acids within the spermatozoa plasma membrane and the low concentration of cytoplasmic scavenging enzymes make these cells highly susceptible to peroxidation in the presence of high levels of ROS in seminal fluid [6–8]. It has been shown that the time of permanence of spermatozoa in the epididymis is longer in oligozoospermic patients, resulting in a higher exposure to ROS [9–11]. The source of ROS in seminal fluid is due to both sperm cells and infiltrating leukocytes [12]. There is also a correlation between leukocyte concentrations, ROS levels, lipid peroxidation, and functional damage [13].

To counteract the potentially damaging effects of oxidative stress (OS), sperm cells and seminal plasma are endowed with some protective antioxidant systems. Spermatozoa have a rather low level of enzymatic antioxidant defence, including catalase, superoxide dismutase (SOD), and glutathione peroxidise. On the contrary, seminal plasma is well endowed with antioxidant buffer capacity [14]. The role of ascorbic acid and uric acid, among the low-molecular-weight antioxidants in seminal plasma, has also been highlighted [14].

Studies in infertile men have shown an impaired seminal plasma nonenzymatic antioxidant capacity [15]. The total oxyradical scavenging capacity (TOSC) is a recently developed assay measuring the overall capability of biological fluids or cellular antioxidants to neutralize the toxicity of various oxyradicals [16, 17]. The TOSC assay can discriminate between different forms of ROS, allowing to identify the role of specific antioxidants in the onset of pathological processes. An early application of the TOSC assay in andrology showed a reduced antioxidant efficiency in seminal fluid of infertile men with a significant correlation between the scavenging capacity of hydroxyl radicals and the parameters of sperm cell motility [18]. Another simpler method to measure the total antioxidant system (TAOS), also called total antioxidant capacity (TAC), was conceived by Rice-Evans and Miller [19]. In this method, the system metmyoglobin-H_2_O_2_ is used as source of radicals, which interaction with the chromogen 2, 2^I^-azinobis-3-ethylbenzothiazoline-6-sulphonate (ABTS) generates a radical cation which is spectroscopically detectable. Antioxidants contrast this reaction, so that the lag time in the formation of ABTS^+°^ is proportional to the antioxidant content. In a 2003 study, for the first time we applied this method, with minor modifications, to seminal plasma from varicocele infertile patients [20].

## 2. Studies with Varicocele Patients

Varicocele (VAR) is a model of male infertility particularly interesting, from the andrological point of view, not only for its prevalence (19–41% of infertile men) [21] but also in relation to an open debate on the real need of surgical or sclerotherapic treatment and on its role in causing infertility. Furthermore, different studies suggest that OS is a common mechanism underlying VAR, as well as X-irradiation, exposure to environmental toxicants, or other physical conditions such as cryptorchidism. All these stress conditions can cause changes in testicular microvascular blood flow and endocrine signalling, eventually leading to germ cell apoptosis and hypospermatogenesis [22]. 

Other studies suggest that VAR patients have high OS also in the case of normozoospermia. DNA fragmentation index (the percentage of sperm cells with denatured DNA), determined by flow cytometry, and the percentage of TUNEL-positive cells, another estimate of sperm DNA damage, have been found significantly greater in VAR, either with normal or abnormal semen profile, than in control fertile subjects. Likewise, ROS levels were significantly higher in both sub-groups of VAR patients [23]. Sakamoto and colleagues showed a significantly higher hexanoyl-l-lysine (HEL) concentration and SOD activity in seminal plasma in azoospermic and oligozoospermic patients; those with VAR had a significantly higher NO, HEL, and SOD activity in plasma. Oligozoospermic patients with VAR presented significantly higher IL-6 in seminal plasma. After varicocelectomy, a significant increase in sperm concentration was found together with a reduction in NO, HEL, 8-Hydroxydeoxyguanosine (8-OHdG) levels, and SOD activity, a reduction in IL-6, and a decrease in the percentage of apoptosis-positive sperm, evaluated by sperm DNA fragmentation [24].

A prospective study, before and after varicocelectomy, was also performed in subfertile patients. The parameters for evaluating OS changes were (a) 4977 bp deletion of mitochondrial DNA in sperm, as detected by PCR, (b) 8-OHdG content in spermatozoa DNA, measured by HPLC electrochemical method, (c) seminal plasma protein thiols and ascorbic acid. Varicocelectomy had a positive effect on seminal parameters in 22/30 patients, with a decrease in 4977 bp deletion of mitochondrial DNA and 8-OHdG levels, and an increase in plasma thiols and ascorbic acid. Interestingly, also in eight patients in whom semen quality did not improve after surgery, a significant decrease in 8-OHdG in sperm DNA and an increase in thiols and ascorbic acid were observed [25].

A standardized semen quality score, proposed by Pasqualotto and colleagues, applying principal component analysis to nine semen characteristics, did not show differences between infertile and fertile subjects with VAR; however, the mean quality score was lower than that in healthy controls. Moreover, the authors showed significant lower ROS-TAC scores in infertile males compared with control subjects, but the scores of non-VAR fertile subjects were not significantly different from those of fertile men with VAR [26]. Therefore, it can be reasonably hypothesized that the fertility potential in VAR can decline due to OS.

A meta-analysis has been published, collecting 23 human studies on the topic of OS in VAR-associated infertility [27], Four studies of these were selected for the similar methods of measuring ROS. The overall estimate showed higher concentrations of ROS and lower total antioxidant capacity in VAR than in controls. In the mentioned paper by Meucci et al. [20], we showed a Lag value (TAC) significantly greater in VAR patients than in non-VAR. But it should be remembered that measuring TAC is different from measuring ROS. Oligospermic VAR patients showed the greatest values of TAC. Lag and sperm motility significantly correlated in VAR normospermic patients. Follicle-stimulating hormone (FSH) showed significant inverse association with Lag in the same group. This partially unexpected results was interpreted as an ineffective utilization of antioxidants in oligospermic VAR, while in normozoospermic VAR the direct correlation between TAC and motility suggested a protective role toward sperm motility. Finally, in the same subgroup, the inverse correlation with FSH suggested a better utilization of antioxidants thanks to the increasing FSH levels, with a compensation with still unknown mechanisms. However, in the light of the last studies, sperm density could be seen as a factor of antioxidant consumption.

Another important antioxidant is the lipophilic molecule Coenzyme Q_10_ (CoQ), also known as ubiquinone, for its ubiquitous presence in animals and different tissues. It is a component of the mitochondrial respiratory chain, therefore potentially involved in spermatozoa function both for energetic and antioxidant properties. We assayed for the first time CoQ levels both in total seminal fluid and in seminal plasma (obtained by centrifugation) [28]. In this original study, conducted on 77 subjects with normal or pathological findings at a standard semen analysis (according to WHO criteria [29]), CoQ levels, measured by HPLC, showed a significant correlation with sperm count and sperm motility. However VAR patients represented an interesting exception; in fact in these patients the correlation with sperm concentration was preserved, whereas the correlation with sperm motility was lacking. Moreover, they showed an increased plasma to total seminal CoQ ratio in comparison with the other subjects. We suggested a possible molecular defect in VAR; a relative deficiency or a defective utilization of CoQ in sperm cells could contribute to the respiratory chain defect reported in spermatozoa of these subjects, where a reduction in O_2_ consumption had been showed [30]. 

In a following study, we also determined CoQ in the cell pellet of spermatozoa, obtained after centrifugation of semen [31]. We found, once again, a differential pattern in VAR and non-VAR subjects. In non-VAR, a higher concentration of CoQ (expressed as ng per million of cells) was present in the spermatozoa of oligo- and asthenozoospermic patients. This relationship was not observed in VAR, who also showed slightly lower intracellular absolute values of CoQ. Since CoQ is involved in the cell defence against free radical damage, a higher intracellular concentration may represent a mechanism of protection for spermatozoa. In VAR patients, this mechanism seems to be defective, leading to higher sensitivity to oxidative damage. The results were confirmed in extended groups of patients, compared to controls, matched with VAR according to seminal parameters (idiopathic oligozoospermia, isolated asthenozoospermia, normal fertile men) [32]. We remarked the significantly higher proportion of CoQ in seminal plasma; as it reflects an interchange between intra- and extracellular compartments, the different distribution in VAR patients could represent a greater sensitivity to peroxidative damage and could suggest its reduced utilization for energy and therefore a defective motility even in patients with normal sperm count. 

**Figure 1:**
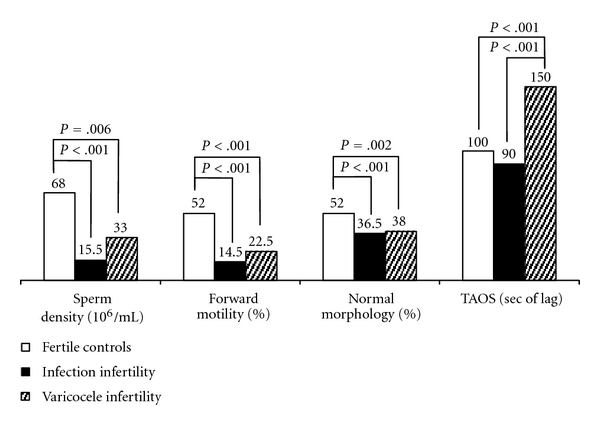
Standard sperm analysis and total antioxidant status (values are medians; data from [33]).

More recently, we studied a group of unselected infertile patients (*n* = 100; divided into 3 subgroups: VAR, infections, other etiologies) and 31 fertile men, also correlating Lag values (TAC) with circulating hormones: gonadotropins, testosterone, estradiol, fT3, fT4, TSH, and prolactin (PRL) [33]. We confirmed the finding of a higher TAC in VAR than controls, while lower values were in patients with inflammation. The regression analysis between hormones and seminal parameters showed an inverse correlation between PRL and sperm motility and a direct correlation of TAC with PRL or fT4, but not with gonadotropins or gonadal steroids. Interesting was the suggestion that systemic hormones may play a role in regulating seminal TAC, even hormones, such as thyroid ones and prolactin, which are not usually tested in the first-level evaluation of male patients with fertility problems.  Figure 1 shows the main results found in that study, with respect to TAC differences in the various groups. 

In another study, a multivariate analysis, including FSH, Lag, percentage of forward-progressive spermatozoa, oligozoospermia, and VAR, had showed a strong inverse correlation between FSH and motility (*r* ^2^ = 0.31, *P* > *F* = 0.0007), not modified by Lag (*r* ^2^ = 0.31, *P* > *F* = 0.002), suggesting that the link between FSH levels and sperm motility is probably represented by the length of Lag phase, suggesting a compensatory role of FSH in modulating antioxidant systems [34].

## 3. Effects of Surgical Treatment

The effect of varicocelectomy on OS, similarly, is not so univocally clear. Vitamin E has been demonstrated to be positively affected by surgical VAR repair [35]. The relationship between varicocelectomy and plasma OS has been studied also in children (10–16 years) with left-sided VAR and ipsilateral testicular hypoplasia, by evaluating basal (presurgery) thiobarbituric acid reactive substances and plasma peroxidation susceptibility (lag time and slope) both in peripheral blood samples and in samples from the pampinous plexus. Peripheral blood samples were re-evaluated 1 year after surgery. Both parameters were comparable in peripheral and pampinous blood and higher compared with controls. After surgery they significantly decreased, again suggesting that surgical varicocelectomy with a venous shunt construction reduces OS. The study highlights that OS is present in children and adolescents with VAR [36].

Our group also studied VAR patients in an attempt to verify whether varicocelectomy had an effect on CoQ distribution in seminal fluid [37]. Only a partial reversion was observed, since the seminal plasma CoQ/total CoQ ratio (higher in VAR vs controls) decreased, but the correlation between total CoQ and motility was not restored; instead, a peculiar correlation between cellular CoQ and motility (an inverse correlation, observed at variance with normal subjects, before surgery) was no longer detectable in postoperative VAR patients.

## 4. Effects of Exogenous CoQ Treatment

The findings mentioned in the previous paragraphs constitute the rationale for treating infertile subjects, particularly VAR, with exogenous CoQ. 

Lewin and Lavon [38] originally reported the effect of CoQ on sperm motility in vitro: a significant increase in motility had been observed in sperm obtained from asthenozoospermic men, incubated with exogenous CoQ, whereas no significant variation was reported in the motility of sperm cells from normal subjects. The same study also reported the effect of exogenous CoQ in vivo, in a group of patients with low fertilization rates, after in vitro fertilization with intracytoplasmatic sperm injection for male factor infertility. No significant changes were reported in most sperm parameters, but a significant improvement was noticed in the fertilization rate after a treatment with CoQ at the dosage of 60 mg/day for a mean period of 103 days.

To investigate a potential therapeutic role, Balercia et colleagues administered CoQ to a group of 22 idiopathic asthenozoospermic infertile patients [39], classified according to WHO-1999 criteria [29]. Patients were given CoQ, 200 mg/day divided into two doses *per os*, for 6 months. Semen analysis, including computer-assisted sperm analysis and motility (C.A.S.A.), CoQ, and phosphatidylcholine assays, were performed at baseline and after 6 months of therapy. A semen analysis was further performed after 6 months from interruption of therapy (wash-out). After treatment, an increase in CoQ and phosphatidylcholine concentrations were found both in seminal plasma and in sperm cells. Regarding semen, a significant difference was found in forward motility of sperm cells after 6 months of CoQ oral supplementation. But the main result was the improvement of sperm motility, also confirmed by means of computer-assisted determination of kinetic parameters (significant increase of VCL and VSL). A positive dependence (using the Cramer's index of association) was evident among the relative variations of seminal plasma or intracellular CoQ content and of C.A.S.A. kinetic parameters (Cramer's V 1/4 0.4637; 0.3818; 0.3467; 0.5148, resp.). A significant reduction in sperm forward motility was reported after 6 months of washout, whereas no significant differences were found in sperm cells concentration and morphology. These results constitute the first demonstration that exogenous administration of CoQ increases its levels in seminal plasma and in spermatozoa. The increment was important, especially in seminal plasma where posttreatment levels were three times higher than basal ones. Similar increases of CoQ concentration (two-threefold higher than baseline value) are commonly found in blood plasma after chronic administration of the quinone [40].

As CoQ is a highly lipophylic molecule, we could reasonably hypothesize its diffusion through the phospholipid bilayer of cellular membranes, but we presently do not know whether transport from blood plasma to testicular and accessory male genital glands is passive or involves an active mechanism. Nevertheless, the good degree of association among these variables, according to Cramer's V index of association, supports the hypothesis of a pathogenetic role of CoQ in asthenozoospermia, according to previously reported data [32]. Apparent improvement of the spontaneous pregnancy rate, even though this was not a primary endpoint of the study, also suggests that this therapeutic approach may be beneficial.

These results were confirmed by a double-blind placebo-controlled clinical trial, realized by the same group [41], using CoQ dosage similar to that used in the previous open trial on male infertility. The study design was: 1 month run in, 6 months of therapy (30 patients) or placebo (30 patients), and a further 3-month followup. The study confirmed the increase in CoQ levels both in seminal plasma and sperm cells; also CoQH (the reduced form of CoQ, with a higher antioxidant efficacy) showed a similar increase. A significant improvement of sperm cell total motility and forward motility was observed in the treated group after 6 months of CoQ administration. The improvement of sperm cell kinetic parameters was also confirmed after computer-assisted analysis, with a significant increase both in VCL and VSL after treatment. Moreover, patients with lower baseline value of motility and lower levels of CoQ had a statistically significant higher probability to be responders to the treatment. Wash-out data confirmed the expected treatment-related results. Nine spontaneous pregnancies were achieved during the observation period. After opening the randomisation list, it was found that six of the patients who had impregnated their female partner had undergone CoQ therapy (three after 4 months, one after 5 months and one after 6 months of treatment). Three pregnancies occurred in partners of patients undergoing placebo treatment: one after 2 months of treatment and the other 2 after 3 months of washout.

A positive effect of CoQ treatment on sperm features was also confirmed in a study by Safarinejad [42], in which 212 infertile men with idiopathic oligoastenospermia were treated either with CoQ (300 mg/day) or placebo. Treatment lasted 26 weeks and was followed by a 30-week washout. Significant improvement in sperm concentration and motility was found with CoQ therapy for both parameters. The Kruger classification sperm morphology revealed an increase in the percent of normal forms in the CoQ group. The CoQ group also showed a significant decrease in serum FSH and LH at 26-week treatment phase. By the end of treatment phase, percentage of acrosome reaction increased in the CoQ group whereas it remained unchanged in the placebo group. Monitoring pregnancy rate was not among the aims of this study.

Taken together, these studies show an improved sperm motility upon exogenous CoQ administration, which could be explained on the basis of the well known involvement of CoQ in mitochondrial bioenergetics and of its widely recognized antioxidant properties. The increased concentration of CoQ in seminal plasma and sperm cells, the improvement of semen kinetic features after treatment, and the evidence of a direct correlation between CoQ concentrations and sperm motility strongly support a direct cause/effect relationship.

## 5. Conclusion

Even if it is clear that Oxidative Stress is present in varicocele as a pathogenic mechanism, however the interpretation of analytical data is not so simple. In fact, antioxidants values can be influenced by different modulatory factors, and anyway they always represent the balance between synthesis and utilization.

A deeper insight into these molecular mechanisms could lead to a greater knowledge of the so-called unexplained infertility. However, different aspects of varicocele physiopathology need to be still investigated, including the age-related effect of oxidative stress, the biochemical composition of seminal plasma.

These data are waiting for being integrated with the novel approaches to infertility studies including genetic and proteomic investigation, which can further clarify to what extent varicocele can affect sperm function and which predictive values we can dispose to better give an indication to surgical or medical therapy in such a condition.
